# The Response of Layer Hen Productivity and Egg Quality to an Additional Limestone Source When Offered Diets Differing in Calcium Concentrations and the Inclusion of Phytase

**DOI:** 10.3390/ani11102991

**Published:** 2021-10-18

**Authors:** Isabelle Ruhnke, Yeasmin Akter, Terence Zimazile Sibanda, Aaron J. Cowieson, Stuart Wilkinson, Stephanie Maldonado, Mini Singh, Patrick Hughes, Dylana Caporale, Stephan Bucker, Cormac John O’Shea

**Affiliations:** 1 School of Environmental and Rural Science, University of New England, Armidale, NSW 2351, Australia; iruhnke@une.edu.au (I.R.); tsiband2@une.edu.au (T.Z.S.); 2 Sydney School of Veterinary Science, University of Sydney, Sydney, NSW 2570, Australia; yeasmin.akter@sydney.edu.au (Y.A.); steph.maldonado@outlook.com (S.M.); mini.singh@sydney.edu.au (M.S.); pjhughes1995@gmail.com (P.H.); dcap0531@uni.sydney.edu.au (D.C.); stephan-buecker@gmx.net (S.B.); 3 DSM Nutritional Products, Wurmisweg, 576 Kaiseraugst, Switzerland; aaron.cowieson@dsm.com; 4 Feedworks, Romsey, VIC 3434, Australia; stuart.wilkinson@feedworks.com.au; 5 School of Life and Environmental Sciences, University of Sydney, Sydney, NSW 2570, Australia; 6 School of Biosciences, University of Nottingham, Loughborough, Leicestershire LE12 5RD, UK; 7 Department of Bioveterinary and Microbial Sciences, Technological University of the Shannon, Midlands Midwest, N37 HD68 Athlone, Ireland

**Keywords:** behavior, choice-feeding, feed, performance, poultry, selection, matrix, minerals

## Abstract

**Simple Summary:**

Dietary calcium is essential for optimal egg production and quality in laying hens, but high concentrations can impede the digestibility of other dietary components. The provision of limestone grit in addition to the main diet may help maintain overall calcium intake while allowing a reduction in dietary calcium levels. The impact of phytase, an enzyme that increases calcium availability in the gut, on the voluntary consumption of limestone grit is unknown. Here, the capacity for hens with access to a separate limestone grit source to modify Ca consumption when offered varying dietary Ca levels and phytase was evaluated. Dietary phytase reduced limestone grit consumption. Egg production was unaffected by reducing dietary calcium levels or the phytase addition. Eggshell measurements worsened in line with decreasing dietary calcium levels despite the provision of limestone grit. In summary, the provision of limestone grit resulted in comparable egg production but not eggshell quality in hens offered suboptimal levels of dietary calcium.

**Abstract:**

Laying hens require substantial quantities of calcium (Ca) to maintain egg production. However, maintaining recommended dietary Ca through inclusion of limestone may impede nutrient digestibility, including that of other minerals. It was hypothesized that providing a separate source of dietary Ca in the form of limestone grit would preserve Ca intake of hens offered diets containing suboptimal Ca concentrations. Furthermore, the impact of dietary phytase at a “superdosing” inclusion rate on the voluntary consumption of limestone grit was evaluated. One hundred and forty-four laying hens (19 weeks of age) were assigned to one of six dietary treatments in a 3 × 2 factorial arrangement comprising three dietary Ca concentrations (40, 30, and 20 g/kg) and ±dietary phytase (3500 FYT/kg diet) on an ad libitum basis for six weeks. Limestone grit (3.4 ± 1.0 mm) was provided to all hens ad libitum. Hens offered diets containing phytase consumed significantly less limestone grit *p* = 0.024). Egg weight, rate of lay, and egg mass were unaffected by dietary treatment (*p* > 0.05). Egg shell weight % (*p* < 0.001), shell thickness (*p* < 0.001), and shell breaking strength (*p* < 0.01) decreased in line with dietary Ca levels. In summary, dietary superdosing with phytase reduced the consumption of a separate limestone source in individually housed, early lay ISA Brown hens. Egg shell quality variables but not egg production worsened in line with lower dietary Ca levels.

## 1. Introduction

Laying hens have a large requirement for dietary Ca to satisfy eggshell synthesis, and this is typically satisfied by dietary inclusion of limestone and other various calcium-containing ingredients. However, dietary limestone is associated with an increase in the pH of avian gastrointestinal tract contents [1,2] which interferes with the enzymatic hydrolysis of digesta. Luminal Ca also binds to plant-derived phytate, forming Ca-phytate complexes which are less susceptible to degradation in the gastrointestinal tract, thus reducing the availability of Ca, P, and various other nutrients [3,4]. Reducing dietary Ca has been one strategy explored by several investigators to lower the quantity of Ca-phytate complexes. However, while reduced Ca intake may not immediately impact egg mass, ultimately, egg and bone composition and mechanical breaking strength can be impaired [5,6].

Providing a choice of diets differing in nutrient profile is a dietary strategy that allows birds to select nutrients based on individual requirements. Holcombe et al. [7] reported how hens preferred high Ca diets when offered a choice. This concept has been extensively reviewed by Rose and Kyriazakis [8] and more recently by Wilkinson et al. [9], and there is rationale to suggest that laying hens are capable of selecting a balanced diet when offered a free choice of nutritionally complementary ingredients. Therefore, offering a separate dietary source of Ca in the form of limestone may preserve an optimal level of total Ca intake to support egg production and quality, albeit obtained partly from dietary Ca and partly from a limestone grit as a separate Ca source. Furthermore, if a specific appetite for Ca exists, the time interval between the consumption of supplementary limestone and consumption of the basal diet may contribute to better utilization of the nutrients contained in the basal diet. However, the ability for poultry to engage in dietary choice is complex and governed by various factors including social behavior, metabolic need, and various other environmental influences [10,11,12], which implies that the ability of hens to exploit feed choice may be limited, or at least not uniform. 

The use of phytase to enhance the availability of phytate-bound minerals including Ca has been widely embraced in the broiler sector, although evidence for improvements in laying hen egg production is lacking and conflicting [13,14]. Nonetheless, phytase has been demonstrated to improve utilization of dietary Ca [14] and presumably enhance luminal Ca concentrations. As extracellular Ca sensing is suspected of being a factor in regulating appetite for nutrients by modulating taste perception [15], the effect of phytase on the ability of hens to modify supplementary Ca consumption is an area which merits investigation, as it may impact current feeding recommendations. 

Therefore, the objective of this study was to evaluate the choice of feed and limestone grit intake, as well as the impact on egg quantity and quality characteristics of hens offered diets with varying dietary Ca concentrations and the inclusion of phytase.

## 2. Materials and Methods

### 2.1. Ethics Statement

The experimental procedures that were conducted in this study were approved by the University of Sydney Animal Ethics Committee (Project Number 2016/945) and were conducted in accordance with the Australian code for the care and use of animals for scientific purposes [16]. 

### 2.2. Experimental Design, Protocol, and Animal Management

A total of 144 ISA Brown hens (17 weeks of age) were housed individually within their cages measuring 25 × 50 × 50 cm with three adjacent cages forming a statistical unit. Hens were habituated to the cages and offered a common point of lay ration for two weeks before experimental treatments were assigned and measurements commenced (19 to 24 weeks of age). At 19 weeks, the rate of lay was 90%. The photoperiod regimen was 16 h of light and 8 h of dark. Each trio of adjacent hens were randomly assigned to one of the six treatment groups, allowing for 8 replicates/treatment spaced randomly throughout the layer house. Diets based primarily on wheat, sorghum, and soybean meal and differing only in dietary Ca (40, 30, and 20 g/kg) and the inclusion or exclusion of phytase (RONOZYME HiPhos; 3500 FYT/kg diet; DSM Nutritional Products Australia Pty Ltd., East Wagga Wagga, Australia) were offered in mash form on an ad libitum basis for a duration of 6 weeks. The ingredient and nutrient compositions of the layer diets are shown in Table 1 and Table 2. The dietary Ca source was primarily from a combination of fine and coarse (~3–4 mm) limestone. The outlined nutrients met all recommended requirements of the ISA Brown laying hen in early lay [17]. All hens were offered ad libitum access to a limestone supplement (average diameter 3.4 ± 1 mm; analyzed 384 g Ca/kg) supplied in an adjacent feeder. All hens were individually weighed at the beginning and end of the experiment using digital scales. Feed and limestone grit usage, and egg production data were collected on a weekly and daily basis, respectively, over the six-week period to calculate the average daily feed intake, limestone grit usage, and laying performance, egg weight, and feed-to-egg conversion ratio (FCR). Once a week, eggs were collected from one bird per trio for egg quality assessment. 

### 2.3. Egg Quality Assessment

During the experimental period, egg quality analysis was conducted once weekly shortly after egg collection on the same day each week. Eggs were allowed to reach room temperature in the laboratory prior to the onset of measurements. Both internal and external egg quality parameters were measured and recorded. Egg weight (g), height (mm), and width (mm) were measured, using a digital scale for weight, to an accuracy of 0.01 g, and a digital caliper (Kincrome, Sydney, Australia) for height and width. Egg height was measured as the length from pole to pole, while width was measured at the equator. Egg breaking force (peak force) was measured using a texture analyzer (TVT 6700 Texture Analyser Perten, Stockholm, Sweden). For internal egg quality testing, the breakout method was employed, using a flat, levelled glass surface on a metal stand with a reflective mirror. Yolk color was determined using a DSM Yolk Colour Fan (DSM, Switzerland), and assigned a value from 1 to 15 units. Using a digital caliper, albumen and yolk width were measured at their widest distance at a 90° angle to each other. Albumen height was measured using a QCD AH reader (Technical Services and Supply Ltd., York, U.K.). Yolk height was measured using a digital height gauge (B.C. Ames Co., Waltham, MA, USA). Albumen and yolk were then separated using a plastic spatula and placed in weigh boats for weighing (g) purposes. Haugh values (1) were calculated using the formula:

Haugh unit = 100 × log (h − 1.7 × w^0.37^ + 7.6) (1)

where h = height of the albumen in millimeters, w = egg weight in grams [18]. 

Eggshell weight was estimated by gently wiping residue albumen from shells with a paper towel and then air-drying for three days at room temperature. Once dried, eggshell weight was measured using digital scales to an accuracy of 0.01 g. Egg shell thickness was measured at three egg segments (top, equator, and base) using a digital caliper (Kincrome, Sydney, Australia) and then averaged. 

### 2.4. Chemical Analysis of Diets, Limestone Supplement, and Excreta

Total excreta were quantitatively collected from each cage, and feed intake was recorded for a 72 h collection period to determine the apparent metabolizable energy (AME) on a dry matter basis, nitrogen (N) retention, and N corrected AME (AMEn). Diets, limestone supplement, and excreta were dried in a forced-air oven at 80 ℃ for 24 h, and the gross energy (GE) of feed and excreta output was determined using a Parr 1281 adiabatic bomb calorimeter (Parr Instrument Company, Moline, IL, USA) which was standardized with benzoic acid and used to determine AME (2) using the following calculation:

AME_diet_ (MJ/kg) = (feed intake (g/day) × GE_diet_ (MJ/kg) − (excreta output (g/day) × GE_excreta_ (MJ/kg)) ÷ feed intake (g/day) (2)

The N content of diets and excreta was determined using an elemental analyzer (Leco Corporation, St Joseph, MI, USA), and N retention (3) was calculated from the following equation:

Coefficient of N retention = (feed intake (g/day) × N_diet_ (g/kg)) − (excreta output (g/day) × N_excreta_ (g/kg)) ÷ (feed intake (g/day) × N_diet_ (g/kg)), (3)

AMEn (MJ/kg) values were calculated by correcting to zero N retention, using the factor of 36.54 kJ/g N retained in the body [19]. The retention of the dry matter (DM; 4) content of the combined intake of diets and the limestone grit was calculated using the following equation:

Coefficient of DM retention = (DM intake of feed + grit (g/day)) − (DM of excreta (g/day)) ÷ (DM intake of feed + grit (g/day))(4)

The mineral composition of the feed, excreta, and limestone grit was determined by inductively coupled plasma optical emission spectrometry (ICP) using a PerkinElmer OPTIMA 7300 (PerkinElmer Inc., Waltham, MA, USA) following digestion with nitric acid and hydrogen peroxide beforehand. The coefficient of Ca retention for combined feed and limestone grit usage and for other feed minerals was calculated using the equations above. 

### 2.5. Statistical Analysis

The data were analyzed as a 2 × 3 factorial arrangement of treatments using the GLM procedure of SAS [20]. The statistical model investigated the main effects of phytase inclusion, calcium concentration, and the associated two-way interactions. If there was a significant interaction, a Tukey’s post analysis was done for multiple comparison. Mean values obtained from a trio of adjacent, individually caged hens served as the statistical replicate for feed intake and egg performance parameters, and eight of those replicates were investigated per treatment. For egg quality assessment and nutrient digestibility assays, one hen of each cage trio was randomly chosen and considered as one replicate. In addition, the lowest and highest 15% limestone users (n = 22) were selected regardless the treatment group for performance parameters comparison using the T-test procedure of SAS [20]. Initial bodyweight was evaluated as a covariate and retained in the model if significant. Statistical differences were reported as significant if *p* < 0.05, <0.01, and <0.001. The development of limestone grit usage, egg weight, rate of lay, and feed intake over time was assessed by repeated measures using the R studio software (v1.3.959; Rstudio, Boston, MA, USA) with the package “rstatix” [21]. Limestone grit usage data was log-transformed prior to statistical analysis. 

## 3. Results

### 3.1. Dietary Composition

The dietary ingredients and calculated and analyzed composition are presented in Table 1 and Table 2. The analyzed Ca content (as is basis) was 48.4 (±3.23), 33.3 (±2.53), and 28.9 (±3.78) g/kg for the 40 g/kg dietary Ca, 30 g/kg dietary Ca, and 20 g/kg dietary Ca treatments, respectively. The average phytase content was 114 and 3695 FYT/kg for the control and phytase-supplemented diets, respectively. The Ca content of limestone grit was 384 g/kg.

### 3.2. Bird Performance and Egg Quality Measurements

**Figure 1:**
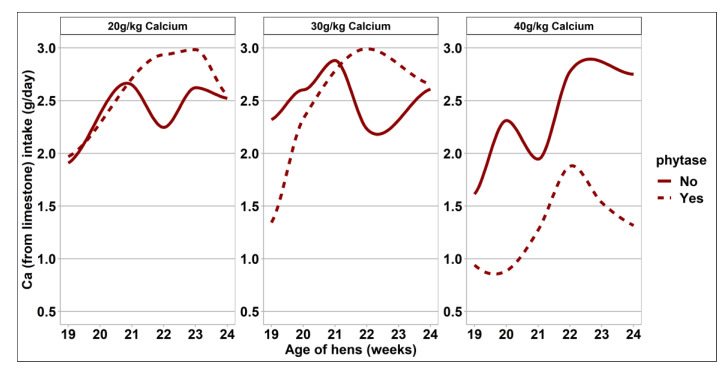
Trendlines of the estimated Ca intake from limestone (grams/hen/day) of hens offered a choice of limestone grit throughout the 6-week experimental period. The data were log-transformed prior to statistical analysis. The data presented here were back-transformed for meaningful visualization (n = 48).

**Figure 2:**
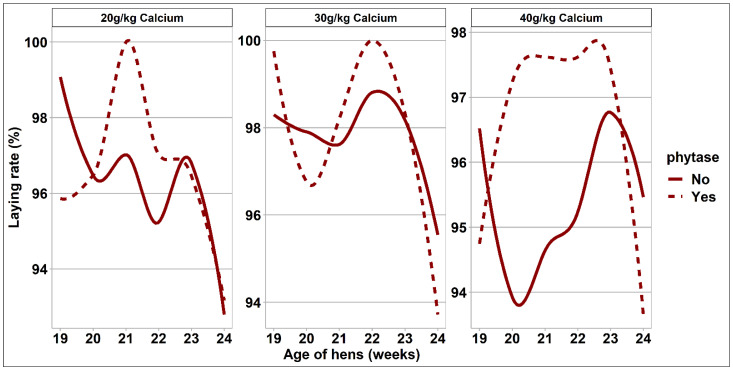
Trendlines visualizing the laying rate that hens of different dietary treatments experienced during the 6-week experimental period (n = 48).

**Figure 3:**
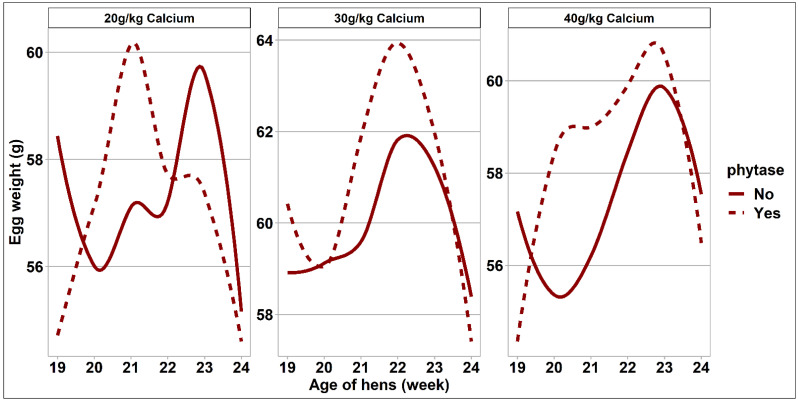
Trendlines visualize the impact and interaction of the calcium level and phytase inclusion of the diet on egg weight during the 6-week experimental period (n = 48).

The response of bodyweight measurements, feed intake, egg output, and feed efficiency to dietary treatments are presented in Table 3 and Figure 1, Figure 2 and Figure 3. There was no difference in bodyweight between treatment groups at the onset of the study. Birds offered the 40 g/kg and 30 g/kg dietary Ca treatments had a greater proportional bodyweight increase and hence a greater final body weight when compared with those of the 20 g/kg Ca diets (*p* = 0.004). Daily feed intake did not differ between treatment groups. However, there was an interaction between phytase and dietary Ca concentrations on calculated daily Ca intake from feed. Daily Ca intake from feed was significantly greater in the 20 g/kg dietary Ca treatment groups supplemented with phytase when compared with complementary Ca diets containing no phytase (*p* = 0.007). However, there was no effect of phytase on daily Ca intake from the 40/g/kg and 30 g/kg dietary Ca group. These differences reflect the measured differences in dietary Ca between phytase inclusion at each Ca level. The overall response of limestone grit intake to dietary treatments is presented in Table 3, and the weekly consumption is presented in Figure 1. Limestone grit usage was affected by dietary phytase inclusion, with birds offered dietary phytase consuming less of the limestone grit when compared with the non-supplemented diets (*p* = 0.024). This effect was the most pronounced in the 40 g/kg Ca diets. The combined intake of Ca from the diet and the limestone grit was affected by dietary Ca level, whereby hens offered the 40 g/kg Ca diets had the greatest total Ca intake, followed by those of the 30 g/kg Ca diets and then the 20 g/kg Ca diets. Phytase inclusion had no effect on total Ca intake. Egg weight, egg mass, and FCR were unaffected by dietary treatments. Overall rate of lay was unaffected by dietary treatment. However, when assessed on a weekly basis, the effect of phytase on laying performance differed among Ca intake groups, where hens which were fed with 40 g/kg Ca and no phytase experienced a higher laying rate (Figure 2). 

To clarify the role of limestone consumption on intake and egg production parameters, the hens were ranked on the basis of whether they were high or low limestone consumers (regardless of dietary treatment group) and compared (Table 4). Initial bodyweight, rate of lay, and egg mass was significantly higher, and dietary Ca intake was lower in the 15% highest limestone intake hens compared to those of the 15% with the least limestone intake.

The response of egg quality measurements to dietary treatments is presented in Table 5. There was no interaction between phytase and Ca concentrations or a main effect of phytase on egg measurements. To further investigate the impact of phytase intake on egg weight separately for each Ca intake group and during the onset of lay, Figure 3 visualizes the (non-significant) interaction using trendlines. Overall, egg weight, yolk weight, and shell weight were the greatest in eggs obtained from hens fed the 40 g/kg Ca diets, which was statistically significant when compared to those of eggs obtained from hens fed the 20 g/kg Ca diet (*p* = 0.030, *p* = 0.010, and *p* = 0.001, respectively). Similarly, the yolk color and eggshell breaking force was the greatest for eggs obtained from hens fed the 40 g/kg Ca diets and statistically significantly different when being compared to those of eggs obtained from hens fed the 20 g/kg Ca diets (*p* = 0.002 and *p* = 0.010, respectively). Shell thickness was the greatest for eggs obtained from hens fed the 40 g/kg Ca diets, intermediate and significantly different for the eggs obtained from hens fed 30 g/kg Ca diets, and thinnest for the eggs obtained from hens fed 20 g/kg Ca diets (all *p* = 0.001). 

### 3.3. Nutrient Digestibility and Retention

The response of measurements of digestibility and nutrient retention are presented in Table 6 and Table 7. There was no effect of dietary treatments on estimates of AME, AMEn, and nitrogen retention. There was a tendency for an interaction between phytase and Ca concentrations on dry matter retention (*p* = 0.084). The addition of phytase to the 40 g/kg dietary Ca increased the coefficient of dry matter retention by 14%. This response to phytase was not observed in the 30 g/kg and 20 g/kg Ca diets. There was an interaction between dietary Ca concentrations and phytase inclusion on Ca retention. Ca retention was lower in the 30 g/kg Ca diet containing phytase when compared with that in the 30 g/kg Ca diet without phytase. The retention of Ca was greater but not significantly different in the 40 g/kg Ca diet containing phytase when compared to that of the same Ca concentration diet without phytase. There was no effect of phytase on Ca retention on hens offered the 20 g/kg Ca diets. There was an interaction between phytase inclusion and dietary Ca concentrations on Cu retention. The retention of Cu was greater for the hens offered the 30 g/kg Ca diet and phytase when compared with that of hens fed the 30 g/kg Ca diet without phytase. However, there was no effect of phytase supplementation on Cu retention at other dietary Ca concentrations. There was an interaction between dietary Ca concentrations and phytase for K retention. K retention was higher for birds offered the 40 g/kg Ca diet and phytase when compared with the that of birds fed the same Ca concentration diet containing no phytase. However, there was no effect of phytase supplementation on K retention of hens offered the 30 g/kg and 20 g/kg Ca diets. Finally, there was a significant effect of dietary Ca concentrations on Fe retention. Fe retention was the greatest for the 20 g/kg Ca diet, intermediate and not different for the 40 g/kg Ca diet, and lowest for the 30 g/kg Ca diet (*p* < 0.05). 

## 4. Discussion

A hypothesis of this study was that reducing dietary Ca and providing supplementary limestone grit may preserve overall Ca intake but with some additional benefits in performance and nutrient retention. The guidelines around Ca nutrition for ISA Brown hens in early lay suggests an ideal concentration of 40–41 g Ca/kg diet with an expected feed intake of between 114–115 g/hen/day, giving an estimated Ca intake of approximately 4.6 g of Ca/hen/day [17]. In this study, all treatment groups exceeded that threshold with hens assigned to the 40 g/kg Ca diets having the highest Ca consumption. Based on these observations, it seems reasonable to assume that Ca intake was adequate for the 20 g/kg dietary Ca group and in line with the intake target for the breed. However, habituation to limestone usage was highly variable; for approximately 40% of the hens, disappearance of limestone grit was less than 1 g/hen/day. In contrast, the top 15% of birds when ranked in terms of greatest limestone intake utilized approximately 7 g/hen/day across all treatments. Habituation of an animal to a feeding practice is a behavior which may be influenced by the actions of other animals within the group [10,22,23]. In this study, the hens were housed individually in wire cages with two dedicated feeders per cage. While a degree of auditory and visual contact was maintained throughout the study, it is unclear how these housing conditions may have impacted the capacity for competitive feeding behavior and can only be speculated [9]. The hens used in this study were at the very early stages of lay, and while the rate of lay was close to the maximum at 97%, bone calcium reserves would be replete at the onset of lay and shortly thereafter [24], which may account for the variable adoption of limestone grit consumption. Feed intake was comparable between groups regardless of Ca concentration or dietary phytase. Phytase inclusion reduced limestone grit usage, and this was particularly pronounced in the hens offered the 40 g/kg Ca diets, where phytase addition resulted in a 49% decrease in grit consumption. Furthermore, this effect of phytase in the highest dietary Ca treatment group was evident from the onset of the study (Figure 2), supporting the theory that Ca appetite specifically may be influenced by the physiological Ca status of the hen as governed by whole body reserves and the quantities of Ca that are available in the digestive tract. The ratio of Ca/phytate was highest in these treatment groups, and releasing this phytate-bound Ca would likely have consequences for Ca-specific appetite. Holcombe et al. [7] reported how 37-week-old layers were able to discriminate between a choice of a Ca-deficient and a Ca-replete diet in order to satisfy metabolic requirements. To account for some of the variability between individuals around limestone grit intake usage, the birds were ranked on the basis of limestone grit intake regardless of dietary assignment. The hens which were ranked as high consumers of the limestone grit consumed 17% less dietary Ca from the basal diet and were heavier from the onset of the study, had a higher rate of lay, a tendency for heavier eggs, and hence a markedly higher egg mass compared with those of the hens ranked as consuming low levels of limestone grit. Extrapolating from these findings, the present study would suggest that some hens were able to redress suboptimal dietary Ca intake with greater utility of a supplementary limestone source, but this nutrient-specific appetite was not uniform. 

Egg production variables such as egg weight, egg mass, rate of lay, and FCR were unaffected by dietary treatments. This suggests that the supplementary limestone consumed in the lower Ca diets preserved these variables. However, caution needs to be exercised in speculating here, as the study focused on early lay birds and only for a six-week period. The hens assigned to the 20 g/kg Ca diets had a lower final body weight. This suggests that these hens were possibly compensating for the undersupply of dietary Ca relative to the other Ca diet groups. Similarly, Ziaei et al. [25] reported that laying hens with reduced calcium intake experienced a reduction in body weight. By using hens at the beginning of lay, the skeletal frame of the average hen would have just finished its development, and providing 20 g/kg of calcium to those individuals might not have been sufficient to promote optimal growth and health [26]. The lower intake of coarse limestone particles from the basal diet might have further compromised nutrient uptake, as coarse feed particles are known to increase organ, especially gizzard, development and benefit nutrient digestibility and uptake via increased intestinal microstructure and transport capacity [27,28]. In contrast, Jiang et al. [5] reported no negative impact on performance variables when 19-week-old layers were fed a 26 g/kg Ca diet, indicating that the 20 g/kg diet fed in the present study might have been just below the minimum requirement and supported an unfavorable Ca/P ratio, regardless of the complementary limestone intake, possibly affecting skeletal Ca metabolism. 

Egg quality variables and particularly those related to shell strength and weight were negatively impacted in birds offered the 20 g/kg Ca diets when compared to those of eggs obtained from hens fed the 40 g/kg Ca diets and, occasionally, eggs obtained from hens fed the 30 g/kg Ca diet. This indicates that even with supplementary limestone grit provision, maintaining adequate Ca intake from the basal diet is still an important objective, particularly when the variability in limestone grit consumption between hens as observed in this experiment is considered. While there was an impact of superdosing with phytase on limestone grit consumption, particularly in the 40 g/kg Ca diet, there was no impact of adding the enzyme on hen body weight gain or any other investigated production parameters. In this study, the levels of non-phytate phosphorus were replete for ISA Browns in early lay, and this may explain the absence of an effect of phytase in improving production parameters. However, Kim et al., [29] reported an improvement in egg production rate of lay, but not for other variables in hens superdosed with phytase at dietary npP levels comparable with those of this study. In contrast, other authors have reported improvements in egg production in response to phytase inclusion, which seems dependent on conditions such as suboptimal non-phytate phosphorus supply [30]. While the level of npP was suitable for all dietary groups, the decreasing Ca concentrations led to a decline in egg productivity and egg quality in the 20 g/kg group, and there is no evidence to indicate that the addition of phytase improved performance in this treatment group in this study. In this group, the lower Ca/npP would be favorable for maximum digestion of both Ca and P, and it therefore seems likely that phytase would be of limited benefit with total dietary Ca supply being the limiting factor.

The inclusion of phytase significantly increased Ca retention in the 40 g/kg Ca diets from 0.529 to 0.740. However, it reduced or did not affect Ca retention in the 30 and 20 g/kg Ca diets. A similar outcome was observed for the retention of dietary K. These interactions are difficult to account for, but nonetheless raise some interesting questions into how high Ca intake and greater Ca bioavailability as effected by phytase may affect voluntary usage of a supplementary limestone grit in laying hens. The review of Selle et al. [4] discussed how higher molar ratios of Ca/phytate-P can result in highly insoluble Ca-phytate complexes which are recalcitrant to enzymatic degradation by phytase. However, in that same review, the findings of Driver et al. [30] were covered, who found that phytase yielded a greater benefit in broilers offered a higher Ca/phytate-P ratio. The response to phytase in laying hens is clearly a topic which merits further study for both its role in nutrient and energy availability and in influencing appetite and feeding behavior. For the retention of other minerals such as Fe and Cu, there were main effects of dietary Ca and an interaction between Ca and phytase, respectively, but the relevance these changes hold for production variables are difficult to unite. Further studies evaluating small intestinal absorption rates may be merited under similar dietary conditions.

## 5. Conclusions

In conclusion, egg production variables were not impacted by lowering dietary Ca level or the addition of phytase in early lay hens offered an additional limestone source. The results of the present study showed that overall limestone grit consumption was not uniform across laying hens. Hence, hens fed the 20 and 30 g/kg Ca diet were not able to compensate Ca intake adequately using the supplementary limestone, resulting in inferior egg quality. In addition, the inclusion of phytase did not impact any investigated performance parameters but decreased limestone intake in hens fed the 40 g/kg Ca diet. Hens can benefit from limestone grit supply when being fed a 40 g/kg Ca diet which will help them to maintain their laying performance, egg quality, and production. 

## Figures and Tables

**Table 1:** Ingredient composition of experimental diets (as fed).

	Dietary Treatments		
Calcium (g/kg Diet)	40	30	20
Ingredients (g/kg)			
Wheat	208	261	314
Soybean meal (48% CP)	266	255	244
Soybean oil	39	24	9.5
Sorghum (9.2% CP)	350	350	350
Limestone	101	74	47
Dicalcium phosphorus	6.9	6.5	6.1
Salt	2.4	2.3	2.3
Sodium bicarbonate	2.5	2.5	2.5
L-Lysine HCl	0.8	1.0	1.1
DL-Methionine	2.2	2.1	2.1
L-Tryptophan	0.3	0.3	0.3
Threonine	0.3	0.3	0.4
Phytase ^1^	(−/+)	(−/+)	(−/+)
Layer premix ^2^	1	1	1
Celite	20	20	20
TOTAL	1000	1000	1000

^1^ DSM Nutritional Products Australia Pty Ltd; RONOZYME HiPhos 3500 FYT/kg diet. ^2^ Provided the following nutrients per kilogram of diet. vitamin A, 10 000 IU; vitamin D, 2500 IU; vitamin E, 25 mg; vitamin K, 2.5 mg; thiamine, 2.5 mg; riboflavin, 5.0 mg; pyridoxine, 3.5 mg; vitamin B12, 0.015 mg; niacin, 30.0 mg; pantothenic acid, 9 mg; folic acid, 1.0 mg; biotin, 0.10 mg; Fe, 60.0 mg; Zn, 60.0 mg; Mn, 50.0 mg; Cu, 5.0 mg; I, 1.0 mg; Co, 0.4 mg; Mo, 0.5 mg; Se, 0.2 mg; apo-ester, 2.9 mg; canthaxanthin, 3.1 mg; ethoxyquin, 25.0 mg (Browning et al. 2015).

**Table 2:** Calculated and analyzed composition of experimental diets (as fed).

		Dietary Treatments		
Calcium (g/kg)	Units	40	30	20
Phytase (−/+)		(−/+)	(−/+)	(−/+)
Calculated				
AME	MJ/kg	11.5	11.5	11.5
Crude protein (N × 6.25)	g/kg	183	183	184
Crude fibre	%	2.1	2.1	2.2
Calcium	g/kg	40.0	30.0	20.0
Phosphorus	g/kg	4.8	4.8	4.8
npP	g/kg	2.5	2.5	2.5
Calcium:npP	Ratio	16.0	12.0	8.0
Chloride	g/kg	2.0	2.0	2.0
Sodium	g/kg	1.7	1.7	1.7
TDC lysine	g/kg	8.5	8.5	8.5
TDC methionine	g/kg	4.4	4.4	4.4
TDC cysteine	g/kg	2.6	2.6	2.7
TDC total sulfur amino acids	g/kg	7.0	7.0	7.1
TDC threonine	g/kg	6	6	6
TDC tryptophan	g/kg	1.9	1.9	1.9
TDC valine	g/kg	7.7	7.7	7.7
**Analyzed**				
Phytase FYT/kg (−/+)		106/3588	99/3436	137/4061
Ca (−/+ phytase)	g/kg	46.0/50.8	33.4/33.2	25.8/32.0
P	g/kg	4.6	4.8	4.8
Crude protein	g/kg	19.9	19.6	20.3
Gross Energy	MJ/kg	15.6	15.8	15.7
Dry Matter	g/kg	91.0	90.7	90.4

(−/+), the addition or not of phytase, AME, Apparent Metabolizable Energy; npP, non-phytate phosphorus; TDC, Total digestible content; FYT, phytase units.

**Table 3:** Effect of differing calcium levels and addition of phytase on bodyweight and feed intake of laying hens (19–24 weeks of age).

Calcium (g/kg)	Phytase	Initial Body Weight (g)	Final Body Weight (g)	Body Weight Change (%)	Feed Intake (g/day)	Ca Intake (from Feed, g/hen/day)	Ca Intake from Limestone (g/hen/day)	Total Ca Intake	Rate of Lay (%)	Egg Weight (g/hen/day)	Egg Mass (g/hen/day)	FCR
40	−	1890	1952	3.29	118	5.43 ^a^	2.45	7.88	98	62	61	1.947
40	+	1857	1930	3.93	117	5.94 ^b^	1.25	7.19	96	62	60	1.955
30	−	1857	1924	3.66	117	3.91 ^c^	2.54	6.45	98	62	61	1.918
30	+	1893	1936	2.27	120	3.99 ^c^	2.50	6.49	98	63	61	1.963
20	−	1854	1859	0.27	116	2.97 ^d^	2.81	5.78	98	61	60	1.940
20	+	1862	1881	1.02	116	3.72 ^c^	2.61	6.33	97	60	59	1.980
SEM		23	25	0.870	2.38	0.099	0.549	0.553	0.704	0.871	0.871	0.040
40		1874	1941 ^a^	3.61 ^a^	117	5.68	1.85	7.53 ^a^	97	62	60	1.95
30		1875	1930 ^a^	2.97 ^a^	119	3.95	2.52	6.47 ^ab^	98	63	61	1.94
20		1858	1870 ^b^	0.64 ^b^	116	3.34	2.71	6.06 ^b^	98	61	59	1.96
SEM		17	18	0.62	1.68	0.070	0.392	0.391	0.498	0.616	0.616	0.028
	−	1867	1911	2.40	117	4.10	2.60	6.70	98	62	61	1.93
	+	1871	1916	2.41	118	4.55	2.12	6.67	97	62	60	1.97
SEM		13	14	0.503	1.37	0.057	0.320	0.320	0.407	0.503	0.503	0.02
*p*−values												
Ca		0.725	0.014	0.004	0.599	0.001	0.196	0.031	0.590	0.128	0.105	0.886
Phytase		0.849	0.842	0.994	0.666	0.001	0.024	0.938	0.345	0.784	0.457	0.344
Interaction		0.349	0.657	0.393	0.635	0.007	0.275	0.532	0.603	0.706	0.670	0.886

Each statistical replicate comprises the mean of 3 individually housed hens and 8 replicates/treatment; FCR, feed-to-egg mass conversion ratio; means with a shared superscript are not significantly different.

**Table 4:** Comparison of the performance data of the extreme 15% of hens ranked as high or low on limestone grit intake, regardless the treatment groups.

Variable	Low Limestone Grit Intake (*n* = 22)	High Limestone Grit Intake (*n* = 22)	SEM	*p*-Value
Limestone grit intake (g/day) *	0.039 (0.013)	6.97 (1.14)	-	-
Initial body weight (g)	1841	1913	23.5	0.038
Final body weight (g)	1920	1911	18.9	0.744
Feed intake (g/hen/day)	114	114	1.86	0.855
Ca intake (from feed, g/hen/day)	4.57	3.91	0.226	0.043
Rate of lay (%)	91	97	1.52	0.024
Egg weight (g/hen/day)	57	61	1.34	0.067
Egg mass (g/hen/day)	53	59	1.87	0.030
FCR	2.01	1.94	0.036	0.182

Twenty-two individual hens selected with the highest and lowest individual limestone grit intake were used for this analysis. * The number in brackets refers to the standard deviation.

**Table 5:** Effect of differing Ca levels and addition of phytase on egg quality of laying hens (19–24 weeks of age).

Calcium	Phytase	Egg Weight (g)	Albumen Height (mm)	Albumen Weight (g)	Haugh Unit	Yolk Height (mm)	Yolk Weight (g)	Yolk Color	Egg Breaking Force (g)	Shell Weight (g)	Shell Weight (%)	Shell Thickness (mm)
40	−	63.5	9.8	37.9	98	16.9	15.4	11.8	4667	6.62	10.4	0.401
40	+	64.2	10.4	39.2	100	17.3	15.4	11.7	4781	6.78	10.5	0.406
20	−	62.4	9.7	37.6	97	17.1	14.9	11.2	4472	6.50	10.4	0.388
20	+	64.3	9.6	38.7	96	16.3	15.3	11.6	4448	6.32	9.9	0.384
30	−	60.9	9.8	37.3	98	17.1	14.9	10.9	4203	5.80	9.4	0.340
30	+	59.3	9.6	35.3	97	18.4	14.3	11.1	4246	5.74	9.7	0.337
SEM		1.13	0.40	0.97	1.79	0.629	0.269	0.180	170	0.17	0.26	0.01
40		63.8 ^a^	10.11	38.6	98.9	17.1	15.4 ^a^	11.7 ^a^	4724 ^a^	6.70 ^a^	10.4 ^a^	0.403 ^a^
30		63.3 ^a^	9.64	38.1	96.9	16.7	15.1 ^a^	11.4 ^a^	4460 ^ab^	6.41 ^a^	10.2 ^a^	0.386 ^b^
20		60.1 ^b^	9.70	36.3	97.7	17.7	14.6 ^b^	11.0 ^b^	4224 ^b^	5.77 ^b^	9.6 ^b^	0.339 ^c^
SEM		0.796	0.282	0.684	1.270	0.445	0.190	0.128	121	0.123	0.181	0.004
	−	62.3	9.79	37.6	97.7	17.1	15.1	11.3	4442	6.31	6.31	0.376
	+	62.6	9.85	37.7	98.0	17.3	15.0	11.5	4495	6.29	6.28	0.376
SEM		0.650	0.231	0.559	1.03	0.34	0.13	0.10	97.9	0.09	0.100	0.003
*p*−values												
Ca		0.003	0.449	0.053	0.555	0.260	0.010	0.002	0.010	0.001	0.006	0.001
Phytase		0.735	0.861	0.883	0.842	0.568	0.784	0.279	0.705	0.874	0.866	0.892
Interaction		0.314	0.496	0.168	0.570	0.265	0.165	0.476	0.917	0.614	0.220	0.737

Means with a shared superscript are not significantly different.

**Table 6:** Effect of dietary Ca concentrations and addition of phytase on measurements of digestibility and retention.

Ca (g/kg)	Phytase	AME	AMEn	N Retention	Dry Matter Retention
40	−	13.5	13.4	0.535	0.648
40	+	13.5	13.3	0.527	0.739
30	−	13.7	13.6	0.501	0.731
30	+	13.7	13.6	0.530	0.695
20	−	13.6	13.5	0.577	0.704
20	+	13.5	13.4	0.587	0.697
SEM		0.304	0.296	0.038	0.029
40		13.5	13.4	0.531	0.694
30		13.7	13.6	0.515	0.713
20		13.6	13.4	0.582	0.700
SEM		0.215	0.210	0.027	0.020
	−	13.6	13.5	0.538	0.694
	+	13.6	13.4	0.548	0.710
SEM		0.176	0.171	0.250	0.017
*p*−values					
Ca		0.785	0.763	0.189	0.789
Phytase		0.831	0.793	0.744	0.495
Interaction		0.983	0.984	0.885	0.084

AME/n, apparent metabolizable energy/corrected for nitrogen retention; N, nitrogen.

**Table 7:** The impact of different dietary Ca levels, phytase, and limestone consumption on measurements of macro and micro mineral retention.

Ca (g/kg)	Phytase	Na	Ca	P	Cu	Fe	Mg	K	Mn
40	−	0.666	0.529 ^ac^	0.447	0.334 ^c^	0.350	0.209	0.304 ^ab^	0.143
40	+	0.710	0.740 ^abc^	0.363	0.239 ^bc^	0.517	0.399	0.435 ^c^	0.386
30	−	0.694	0.777 ^ab^	0.357	0.060 ^b^	0.434	0.402	0.308 ^ab^	0.182
30	+	0.664	0.551 ^c^	0.232	0.338 ^c^	0.364	0.338	0.279 ^b^	0.192
20	−	0.674	0.849 ^b^	0.406	0.378 ^ac^	0.537	0.349	0.405 ^ac^	0.313
20	+	0.689	0.821 ^b^	0.389	0.497 ^a^	0.486	0.406	0.363 ^abc^	0.319
SEM		0.024	0.085	0.057	0.053	0.072	0.079	0.037	0.055
40		0.688	0.634	0.405	0.286	0.433	0.304	0.369	0.265
30		0.679	0.664	0.294	0.199	0.399	0.370	0.294	0.187
20		0.682	0.835	0.398	0.438	0.511	0.377	0.384	0.316
SEM		0.017	0.060	0.041	0.038	0.051	0.056	0.026	0.040
	−	0.678	0.718	0.403	0.257	0.440	0.320	0.339	0.213
	+	0.688	0.704	0.328	0.358	0.456	0.381	0.359	0.299
SEM		0.014	0.049	0.033	0.031	0.042	0.046	0.021	0.032
*p*−values									
Ca		0.919	0.064	0.130	0.001	0.285	0.595	0.038	0.093
Phytase		0.611	0.840	0.117	0.029	0.790	0.355	0.515	0.072
Interaction		0.301	0.044	0.645	0.007	0.222	0.289	0.045	0.071

Ca, calcium; Na, sodium; P, phosphorus; Cu, copper; S, sulfur; Fe, iron; Mg, magnesium; K, potassium; Mn, manganese. Means with a shared superscript are not significantly different.

## Data Availability

Data is available upon request from the corresponding authors.
